# Supplementary material for: Quantification and statistical modeling of droplet-based single-nucleus RNA-sequencing data
Source: Biostatistics. 2023 May 31;25(3):801–17. doi: 10.1093/biostatistics/kxad010 (PMC11247185; doi:10.1093/biostatistics/kxad010)
Supplement: kxad010_Supplementary_Data [file kxad010_supplementary_data.zip › biosts-22175-File011.pdf]

Supplementary Materials

Quantification and statistical modeling of droplet-based single-nucleus RNA-sequencing data

Albert Kuo, Kasper D. Hansen, Stephanie C. Hicks*

*Correspondence to shicks19@jhu.edu

Contents

1. **Supplemental Notes.**
2. **Supplemental Figures S1-S13.**
3. **Supplemental Tables S1-S2.**

Supplemental Notes

Note 1: Description of methods for distribution plots

We first remove the major sources of biological variation by subsetting the nuclei by cell type and biological replicate (**Figure S2**) in order to obtain a pseudo-negative control dataset. Each nucleus $\times$ gene matrix subset is then downsampled to remove variability due to differences in sequencing depth and obtain comparable library sizes across nuclei.

Given a downsampled $m \times n$ matrix M_t with m genes and n nuclei of a given cell type t, we calculate the following values. Let x_{ij} be the number of reads for gene i and nuclei j. For every gene i, the empirical mean is defined as $\bar{x}_i = \frac{\sum_{j=1}^{n} x_{ij}}{n}$, the empirical variance is defined as $s_i^2 = \frac{\sum_{i=1}^{n}(x_{ij}-\bar{x}_i)^2}{n}$, and the empirical probability or fraction of zero droplets is given by $P(x_i = 0) = \frac{\sum_{j=1}^{n} \mathbb{1}(x_{ij}=0)}{n}$.

The theoretical variances and probability of zero droplets is computed for each distribution using parameters estimated from the data. To estimate the parameters for a binomial distribution, $X_i \sim Binom(n, p_i)$, let $\hat{n}$ be the median column sum of M_t and $\hat{p}_i = \frac{\sum_{j=1}^{n} x_{ij}}{\sum_{i=1}^{m}\sum_{j=1}^{n} x_{ij}}$. For a Poisson distribution, $X_i \sim Poisson(\lambda_i)$, let $\hat{\lambda}_i = \hat{n} * \hat{p}_i$. For a negative binomial (NB) distribution with an overall dispersion parameter, $X_i \sim NB(\phi, \mu_i)$, where ϕ is the dispersion parameter and μ_i is the mean, let $\hat{\mu}_i = \hat{n} * \hat{p}_i$. To estimate $\hat{\phi}$, note that $\sigma^2 = \mu + \mu^2/\phi$. Therefore, using the empirical means and variances for every gene i, we can estimate $\hat{\phi}$ as the maximum likelihood coefficient from the following linear regression: $s_i^2 = \bar{x}_i + \bar{x}_i^2/\phi$. For a negative binomial distribution with gene-specific dispersion parameters, $X_i \sim NB(\phi_i, \mu_i)$, let $\hat{\mu}_i = \hat{n} * \hat{p}_i$. We estimate $\hat{\phi}_i$ as the maximum likelihood estimate of a generalized linear model $g(E(X_i)) = \beta_0$ with the negative binomial family, where each observation is a different nucleus and a separate model is estimated for every gene using the `mgcv` package [11]. This is what we refer to as the gene-specific (G-S) negative binomial distribution. After the parameters for each distribution have been estimated, we can compute the theoretical variances and probability of zero droplets, $P(X_i = 0)$, under each distribution. We also calculate the log-likelihood (LL) under each distribution for each gene.

The BIC log-likelihoods are computed using the formula BIC $= k\log(n) - 2\log(L)$, where k is the number of parameters, n is the number of observations, and L is the maximum likelihood [6]. The BIC is calculated using the log-likelihood of the sum across all genes (assuming independence of genes) and observations. Code used to perform these computations is adapted from [7] and all plots are generated using the `ggplot2` R package [9].

For the goodness-of-fit tests, a Pearson's chi-squared statistic was computed for every gene i. The formula for the Poisson distribution is given by $X^2 = \sum_j \frac{(x_{ij}-\hat{\mu}_{ij})^2}{\hat{\mu}_{ij}}$, where the sum is over nuclei of a given cell type and biological replicate. $\hat{\mu}_{ij}$ is the maximum likelihood estimate of the Poisson mean, and is given by $\hat{\mu}_{ij} = \hat{c}_j\hat{\lambda}_i$, where $\hat{c}_j = \sum_i x_{ij}$ is the column sum for cell j and $\hat{\lambda}_i = \frac{\sum_j x_{ij}}{\sum_j \sum_i x_{ij}}$ is the empirical rate at which reads maps to gene i. If the counts x_{ij} are independent and follow a Poisson distribution with mean μ_{ij}, then the statistics follow a chi-squared distribution with $n-1$ degrees of freedom [4].

When μ_{ij} is small ($\mu_{ij} <\approx 1$), as is often the case with snRNA-seq counts, the distribution of the chi-squared statistics is not well-approximated by the chi-squared distribution [10]. We found this in our application of snRNA-seq data as well (**Figure S13a**), where we ran the goodness-of-fit test on counts simulated from a Poisson distribution. We plot the quantile-quantile plots from a Poisson goodness-of-fit test for five different counts matrices, each with 21483 rows and 347 columns, which roughly corresponds to the number of genes (rows) and nuclei (columns) we encounter in our snRNA-seq dataset after restricting to a given cell type and cortex. Each counts matrix follows a Poisson distribution with a different mean parameter μ, ranging from 0.1 to 1.0. We observe that as μ decreases, the chi-squared statistics from the goodness-of-fit test increasingly deviate from the theoretical quantiles of a chi-squared distribution, which indicates that directly applying such a test to sparse counts matrices with low means is not a reliable test to assess the distributional fit.

To address this, we use grouped chi-squared tests following the method proposed by [10]. We first remove genes whose counts are too sparse and the number of cells we would need to group is more than what is available in our data. For the remaining genes, we use a grouped version of goodness-of-fit tests, where we first group the counts of r nuclei [10]. Let $y_{ik} = \sum_j x_{ij}$ be the sum of the counts of the r nuclei in the kth group and let $\hat{\mu}_{ik}$ be the corresponding empirical mean for y_{ik}. Since the sums of independent Poisson are also Poisson distributed, the chi-squared statistic follows a similar formula, $X^2 = \sum_k \frac{(y_{ik}-\hat{\mu}_{ik})^2}{\hat{\mu}_{ik}}$, and is approximated by a chi-squared distribution with $n_k - 1$, where n_k is the number of groups. We show that by applying this grouping procedure to simulated counts from a Poisson distribution, we get the expected results from the goodness-of-fit test (**Figure S13b**).

Likewise, the chi-squared statistics for the negative binomial distribution is $X^2 = \sum_k \frac{(y_{ik}-\hat{\mu}_{ik})^2}{\hat{\mu}_{ik}+\hat{\mu}_{ik}/\hat{\phi}_i}$. Note that by taking the sum of counts, we are increasing the means $\mu_{ik} > \mu_{ij}$, but reducing the degrees of freedom, to improve the approximation to the chi-squared distribution.

To determine the size of the group r, we choose $r = \frac{1}{2\hat{\mu}_{\min} p}$, where $\hat{\mu}_{\min} = \min_i \left(\frac{\sum_i \hat{\mu}_{ij}}{n} \right)$, the smallest average empirical mean across genes, and $p = 0.25$. This ensures that the component variance of the chi-squared statistic is, on average, no larger than $2(1 + p)$, where 2 is the true theoretical variance of a χ^2_1 distribution.

For visualization purposes, the limits of the axes of the quantile-quantile plots are fixed to constant values across different cell types and reference transcriptomes.

Supplemental Figures

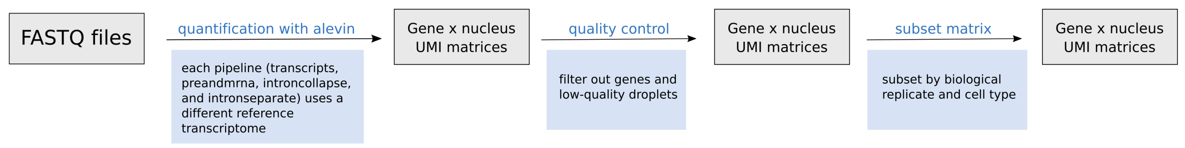

Supplementary Figure S1. Overview of computational workflow to create pseudo-negative control snRNA-seq data. Raw sequencing reads (FASTQ files) from $N = 2$ mouse mouse cortices [2] were processed with `salmon alevin` to perform quantification mapping with four ways for how to include exonic and intronic regions in a reference transcriptome, referred to as (i) *transcripts*, (ii) *preandmrna*, (iii) *introncollapse*, or (iv) *intronseparate* (see **Table S1** for details). This results in four matrices of unique molecular identifier (UMI) counts with genes along the rows and nuclei along the columns. Next, we apply quality control metrics to filter out low-quality nuclei and lowly expressed genes. Finally, we stratify the nuclei by cell type ($C = 7$) and biological replicate ($N = 2$). Pseudo-negative control data represents analyses performed on these stratified subsets where we expect less biological variation within a cell type than across cell types.

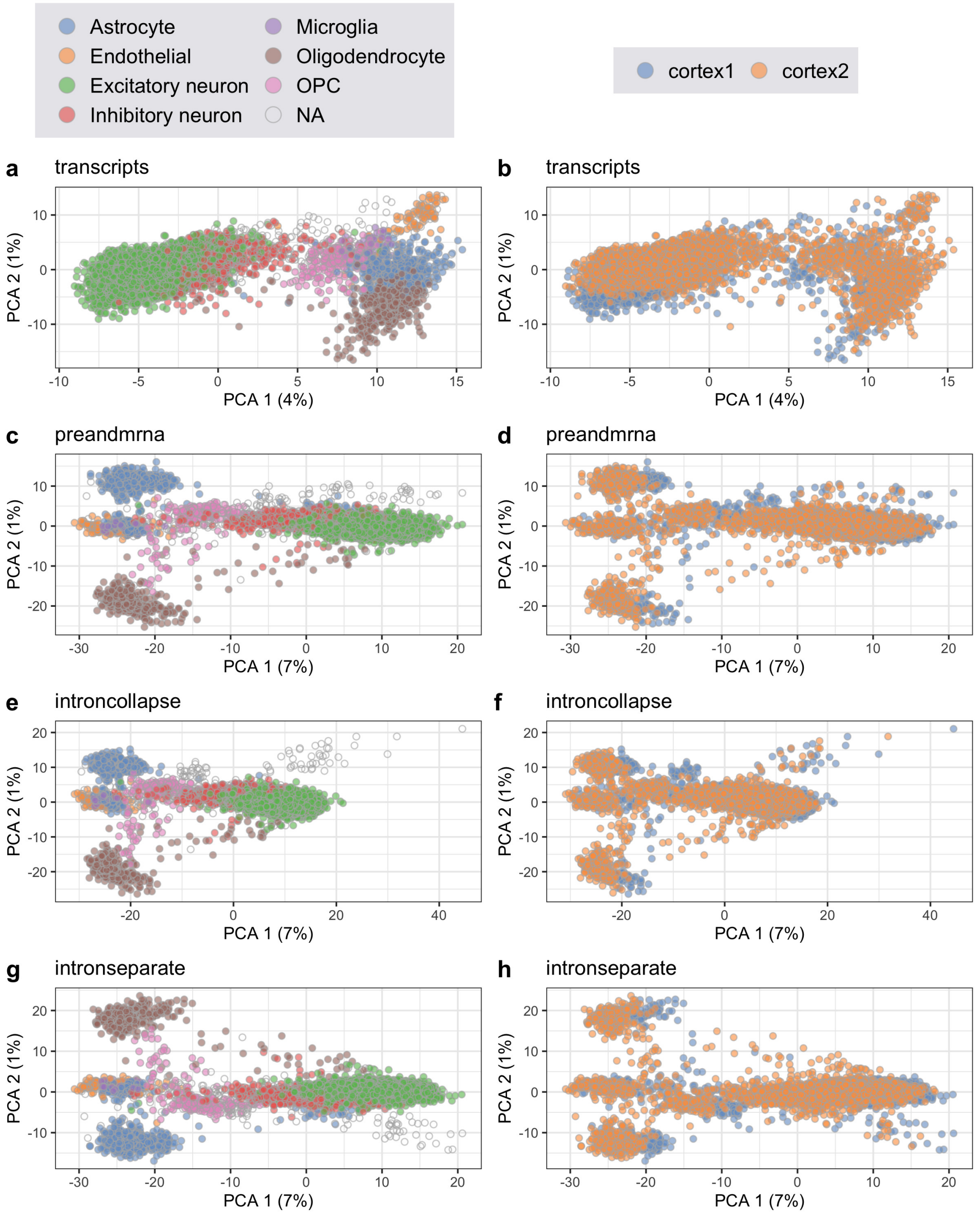

Supplementary Figure S2. Principal components analysis to identify sources of biological variation among nuclei. The first two principal components from principal component analysis (PCA), which was performed on the normalized log-transformed counts using each reference transcriptome (rows). In the left column, we observe that in all references, the main source of variation is explained by the different cell types (colors are cell type labels as classified by [2]). In the right column, we also observe some minor variation by biological replicates (mouse cortices). **(a, b)** *transcripts* reference **(c, d)** *preandmrna* reference **(e, f)** *introncollapse* reference **(g, h)** *intronseparate* reference.

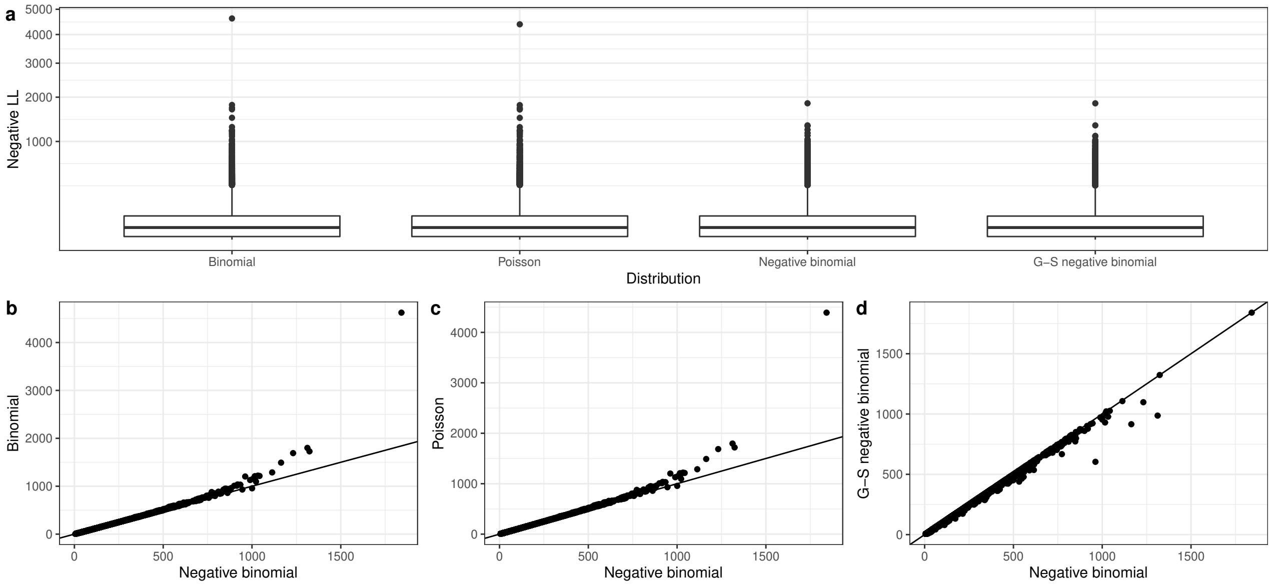

Supplementary Figure S3. Assessment of probability distribution fits for each gene using negative log-likelihoods. Using inhibitory neurons from cortex 1 and the *preandmrna* reference, for each gene (a dot in either the box plot or the scatter plot), the negative log-likelihood (LL) under each distribution is calculated and compared. **(a)** Box plots of the negative log-likelihoods for each distribution **(b)** The negative log-likelihood for every gene under the negative binomial distribution (x-axis) versus the binomial distribution (y-axis) **(c)** The negative log-likelihood for every gene under the negative binomial distribution (x-axis) versus the Poisson distribution (y-axis) **(d)** The negative log-likelihood for every gene under the negative binomial distribution (x-axis) versus the negative binomial distribution with gene-specific overdispersion parameters (y-axis). For each gene (black dot), if the negative LL fit is the same for each distribution, we expect it to fall along the black line (y=x).

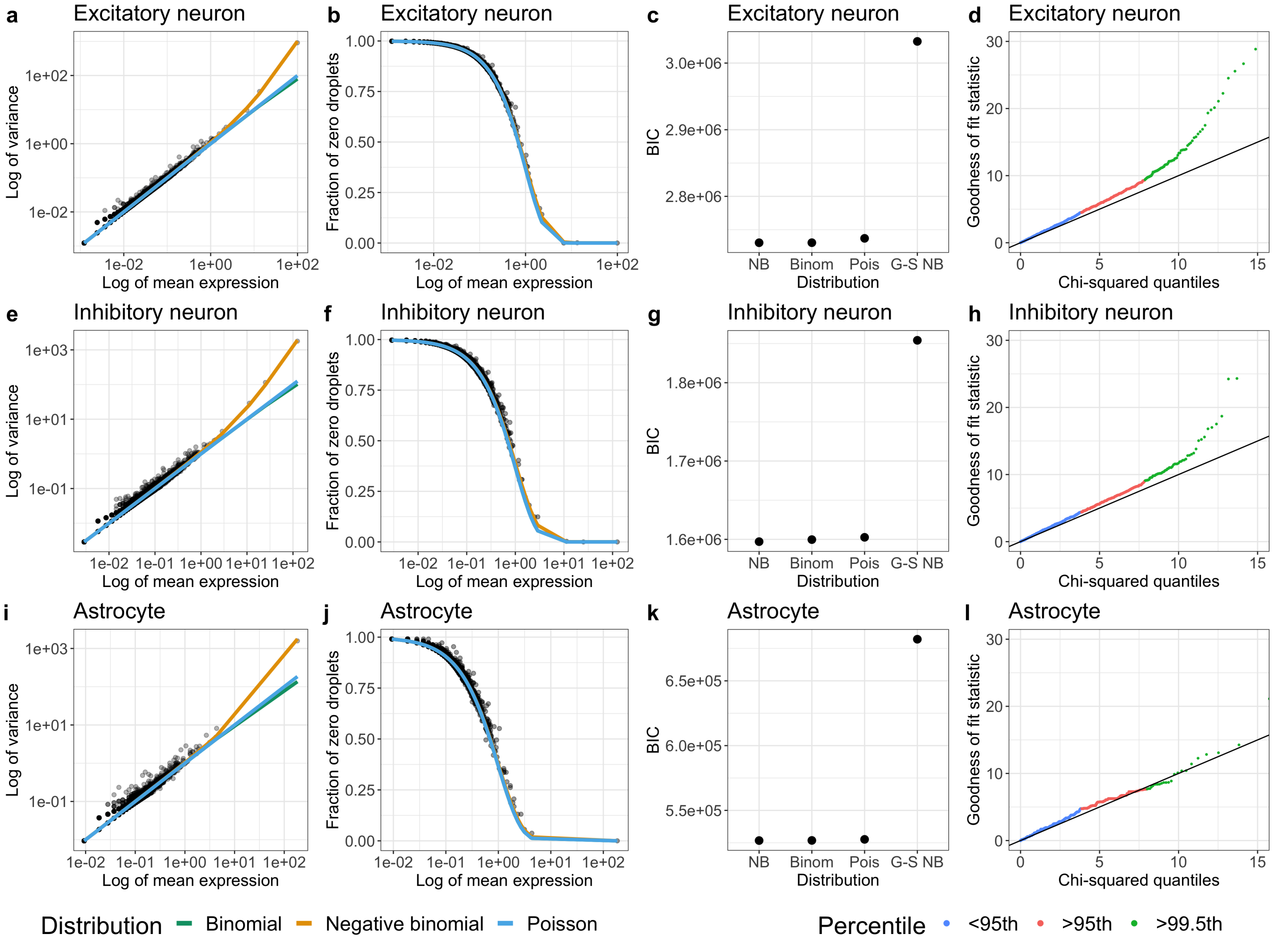

Supplementary Figure S4. Droplet-based scRNA-seq data is not zero-inflated using the 'transcripts' reference. Similar to Figure 1 with subsets of cell types from Cortex 1, but using the *transcripts* reference transcriptome in the quantification mapping tool. **(a-d)** Excitatory neurons **(e-h)** Inhibitory neurons **(i-l)** Astrocytes.

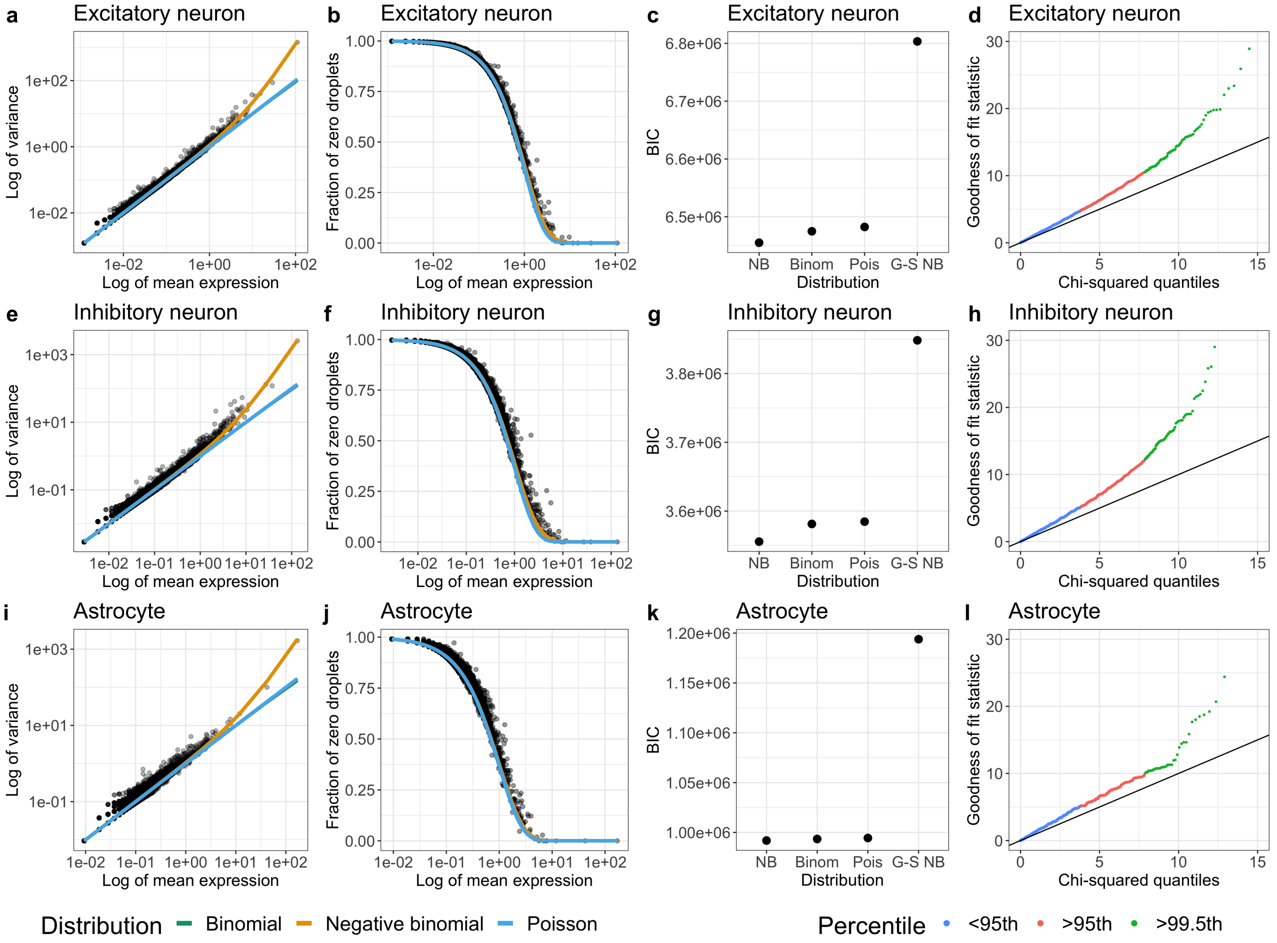

Supplementary Figure S5. Droplet-based scRNA-seq data is not zero-inflated using the 'introncollapse' reference. Similar to Figure 1 with subsets of cell types from Cortex 1, but using the *introncollapse* reference transcriptome in the quantification mapping tool. **(a-d)** Excitatory neurons **(e-h)** Inhibitory neurons **(i-l)** Astrocytes.

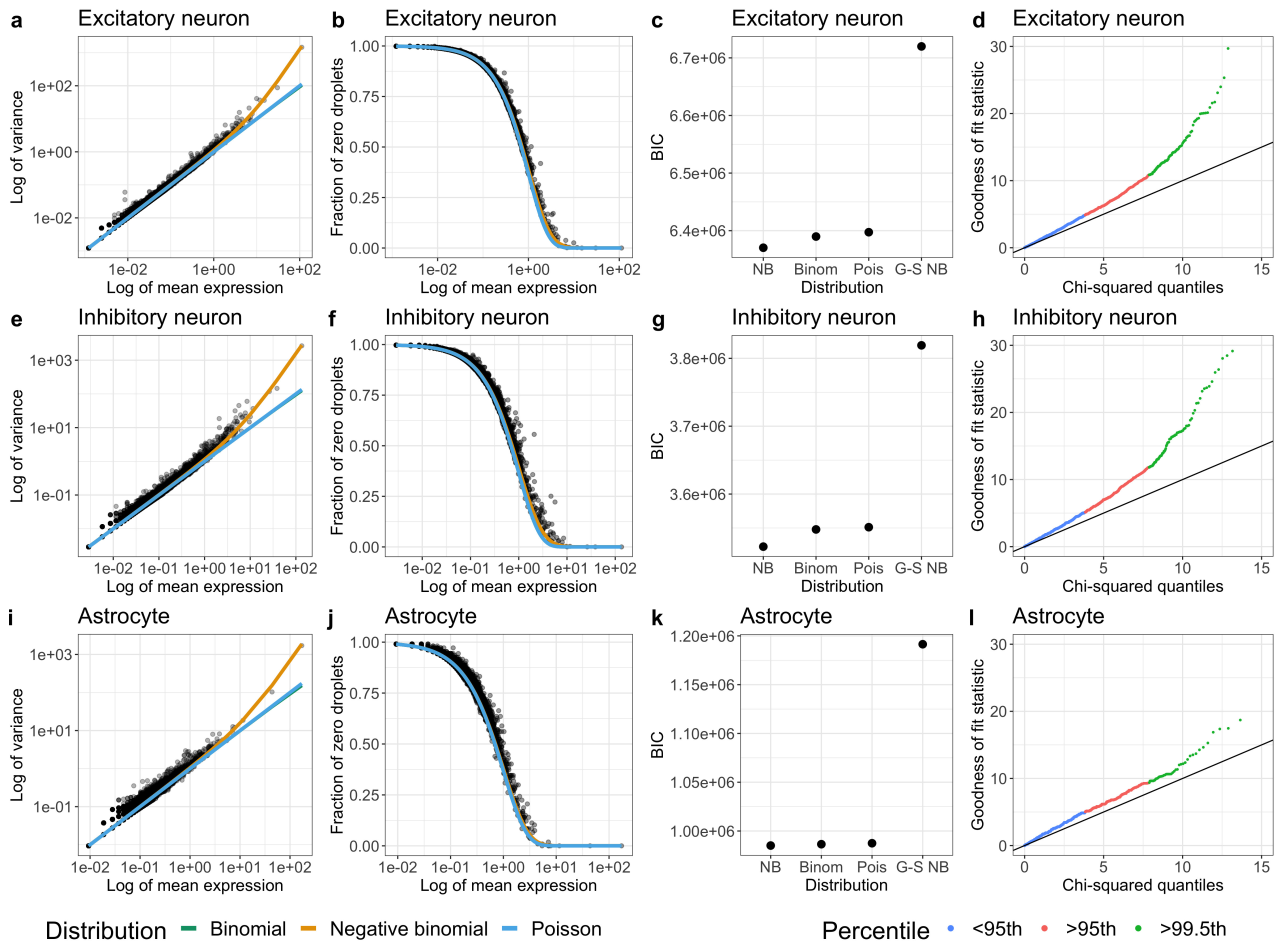

Supplementary Figure S6. Droplet-based scRNA-seq data is not zero-inflated using the 'intronseparate' reference. Similar to Figure 1 with subsets of cell types from Cortex 1, but using the *intronseparate* reference transcriptome in the quantification mapping tool. **(a-d)** Excitatory neurons **(e-h)** Inhibitory neurons **(i-l)** Astrocytes.

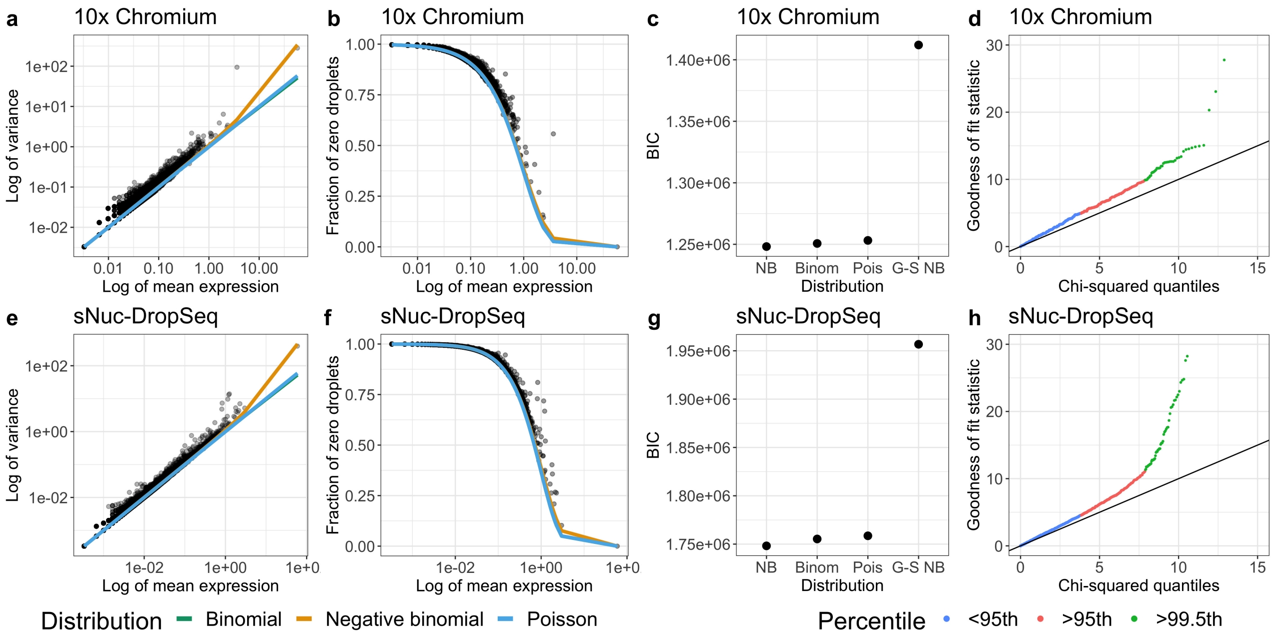

Supplementary Figure S7. Droplet-based snRNA-seq data is not zero-inflated. Results are shown for two additional snRNA-seq datasets, **(a-d)** excitatory neurons cluster (32285 expressed genes × 222 nuclei) from a 10x Chromium mouse brain dataset [1] and **(e-h)** endothelial cells cluster (19713 expressed genes × 305 nuclei) from a sNuc-Dropseq mouse kidney dataset [12]. Clustering and marker gene detection was performed using the `scran` package [3] and clusters were mapped to cell types using the SNAP25 gene for excitatory neurons [8] and the EGFL7 gene for endothelial cells [5]. The leftmost column shows the log-transformed empirical mean (x-axis) and variance (y-axis) for each gene (black dots) with the theoretical variance (colored lines). The second column shows the log-transformed empirical mean (x-axis) and observed fraction of zeros (y-axis) for each gene (black dots) with the expected fraction of zeros under each distribution (colored lines). The third column shows BIC value across all genes (assuming the genes are independent) for each distribution: negative binomial (NB) with one overdispersion parameter estimated for the dataset, binomial (Binom), Poisson (Pois), and NB with gene-specific overdispersion parameters (G-S NB). The rightmost column shows the quantile-quantile plot under the Poisson model with the theoretical chi-squared quantile on the x-axis and the observed chi-squared statistic on the y-axis.

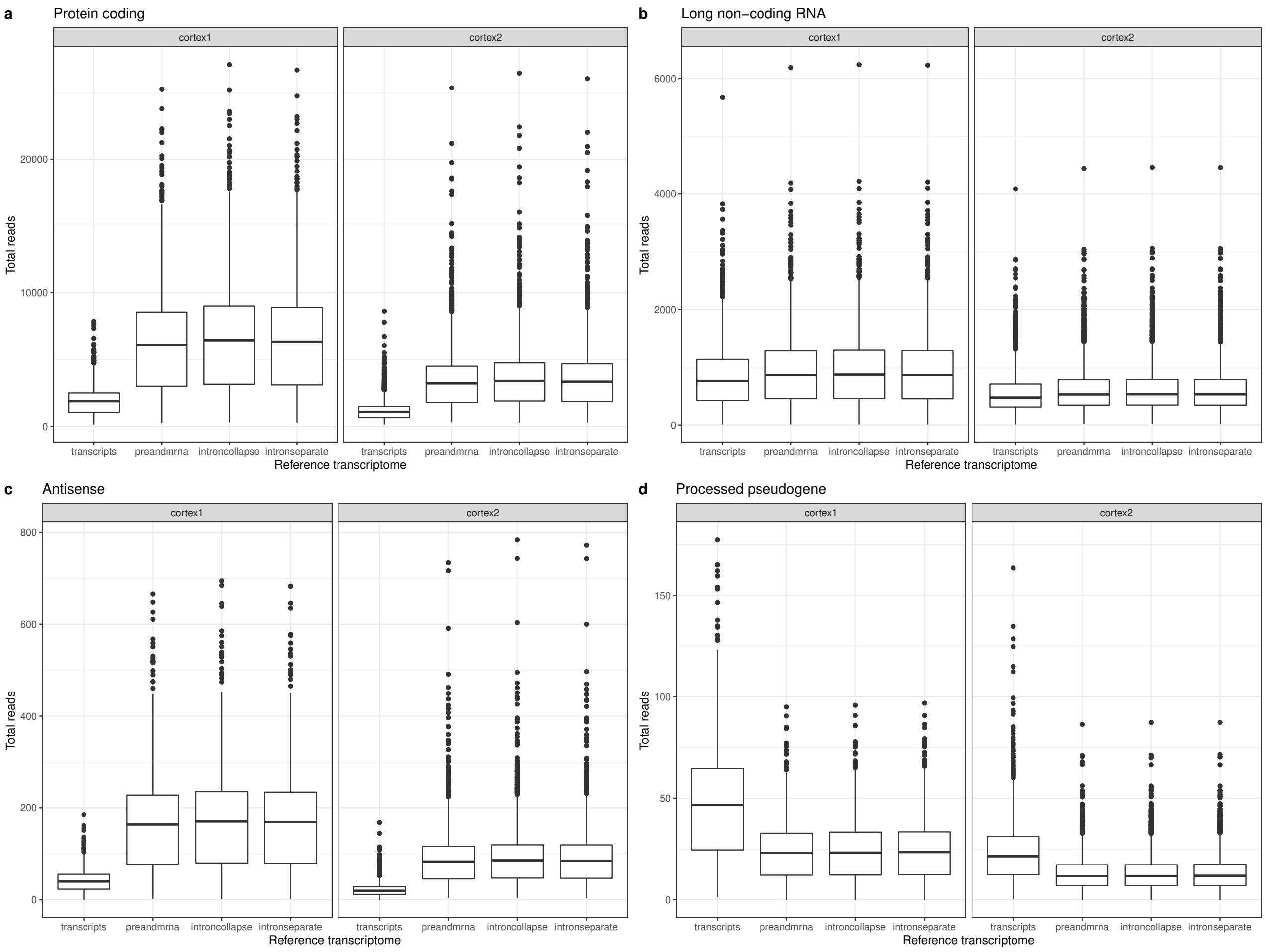

Supplementary Figure S8. Number of mapped reads to gene sets stratified by reference transcriptomes. Similar to Figure 2, but now showing boxplots of the number of mapped reads to **(a)** protein coding genes, **(b)** long non-coding RNA, **(c)** antisense, and **(d)** processed pseudogene. For each gene type, the boxplots are faceted by the two biological replicates (Cortex1 and Cortex2).

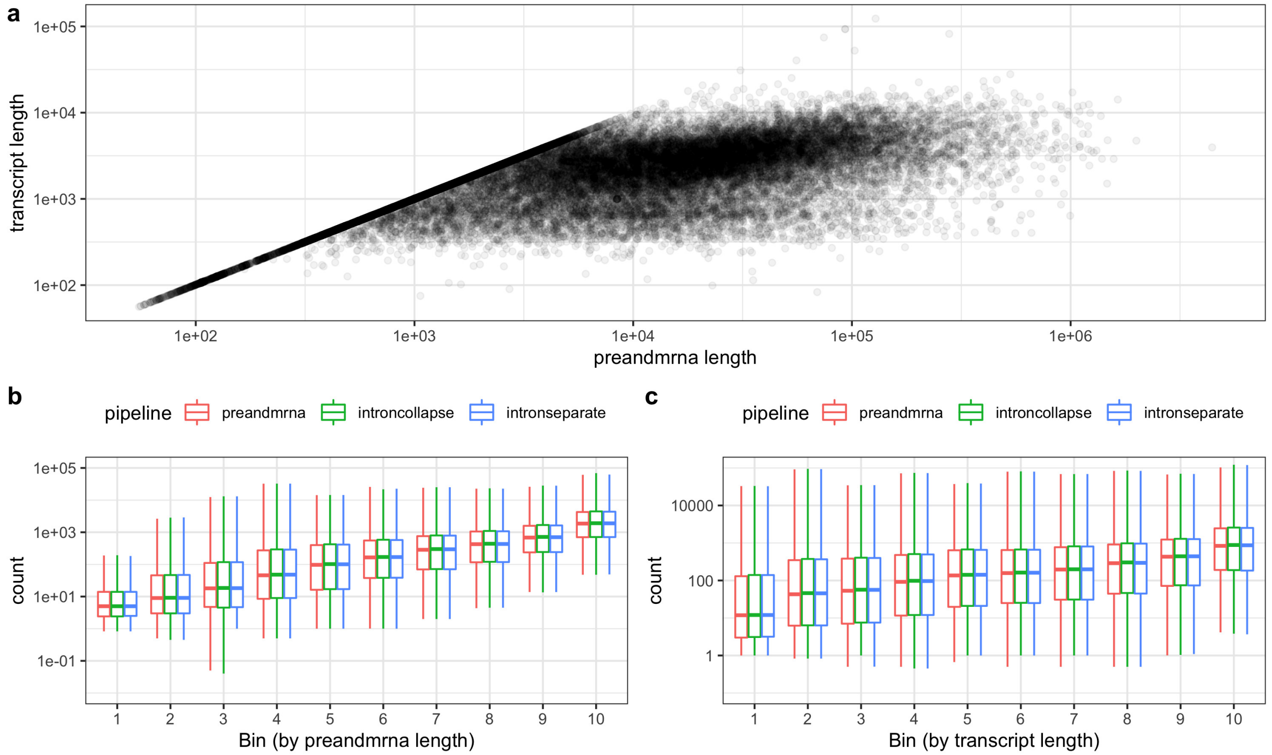

Supplementary Figure S9. Correlation between preandmrna and transcript length and comparison of gene length bias across references with intronic reads. (a) For every gene, the preandmrna length is plotted on the x-axis and the transcript length is plotted on the y-axis. **(b-c)** A similar gene length bias is observed across the three references with intronic regions (outliers not included for boxplots). The sum of counts across all nuclei is plotted on the y-axis and genes are binned into ten equally-sized bins by their **(b)** preandmrna length or **(c)** transcript length.

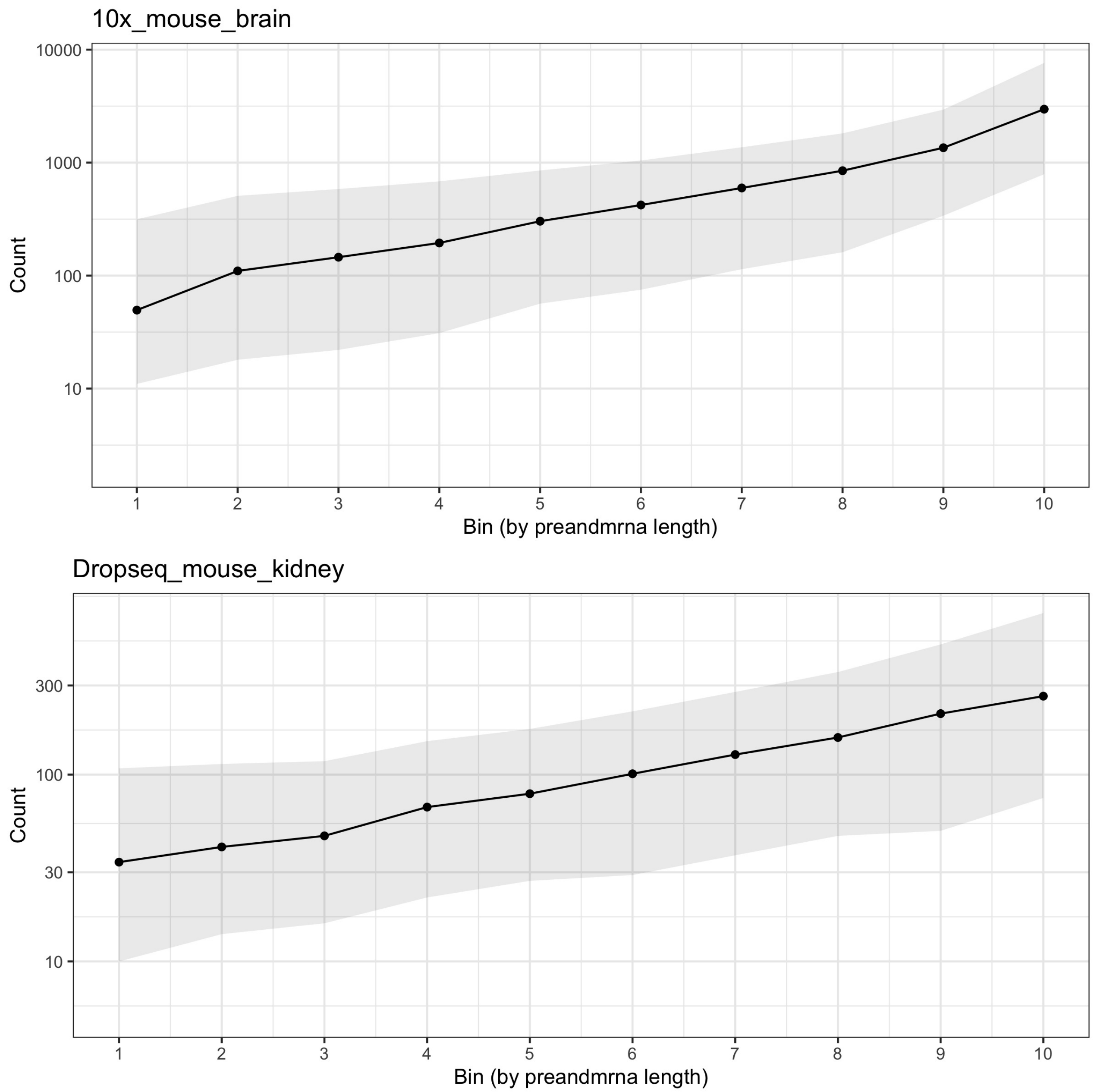

Supplementary Figure S10. Gene length bias in other snRNA-seq datasets. Genes are binned by their 'preandmrna length' (full gene length with both exons and introns) into ten equally-sized bins (x-axis) where the smallest bin number corresponds to the shortest genes and the largest bin number corresponds to the longest genes. Within each bin, the distribution of gene counts across nuclei (y-axis) is shown with the median (black solid points) and the area between the 25th and 75th percentile is shaded in grey. The gene length bias is shown for two external datasets: mouse brain nuclei sequenced with 10x Chromium (top plot) and mouse kidney nuclei sequenced with Drop-seq (bottom plot).

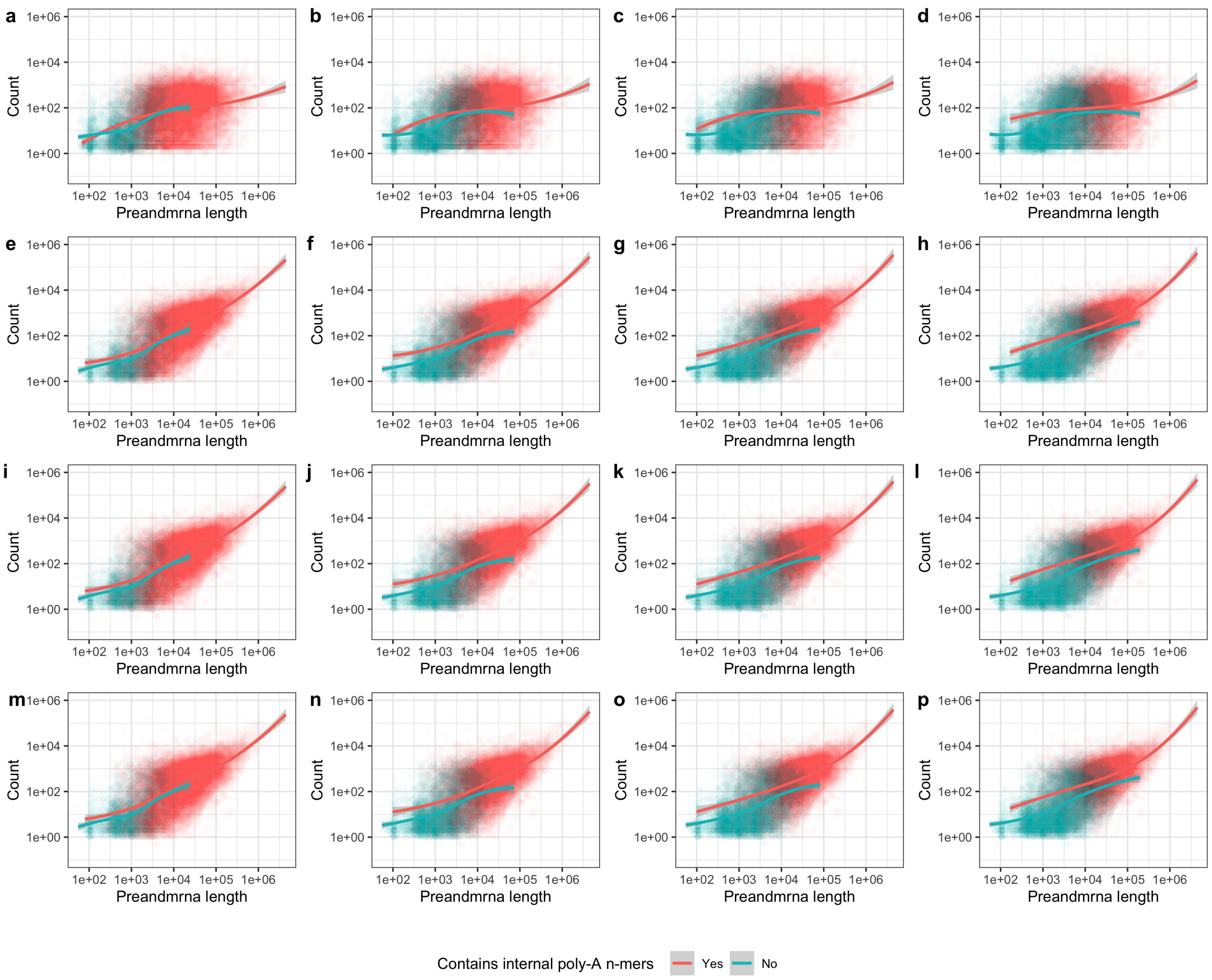

Supplementary Figure S11. Comparison of gene length bias for genes with and without internal poly-A sequences under different reference transcriptomes and n-mer cut-offs. Each point is a different gene, with the sum of counts across all nuclei plotted on the y-axis (base-10 log scale). Genes are colored red if they have at least one internal poly-A n-mer and blue if they do not. A loess curve is drawn for each set of genes. The x-axis uses the full gene length with both exons and introns ('preandmrna' gene length). Each column corresponds to a different poly-A n-mer cut-off **(a, e, i, m)** poly-A 6-mer, **(b, f, j, n)** poly-A 8-mer, **(c, g, k, o)** poly-A 10-mer, **(d, h, l, p)** poly-A 12-mer. Each row corresponds to a different reference transcriptome **(a, b, c, d)** *transcripts*, **(e, f, g, h)** *preandmrna*, **(i, j, k, l)** *introncollapse*, **(m, n, o, p)** *intronseparate*.

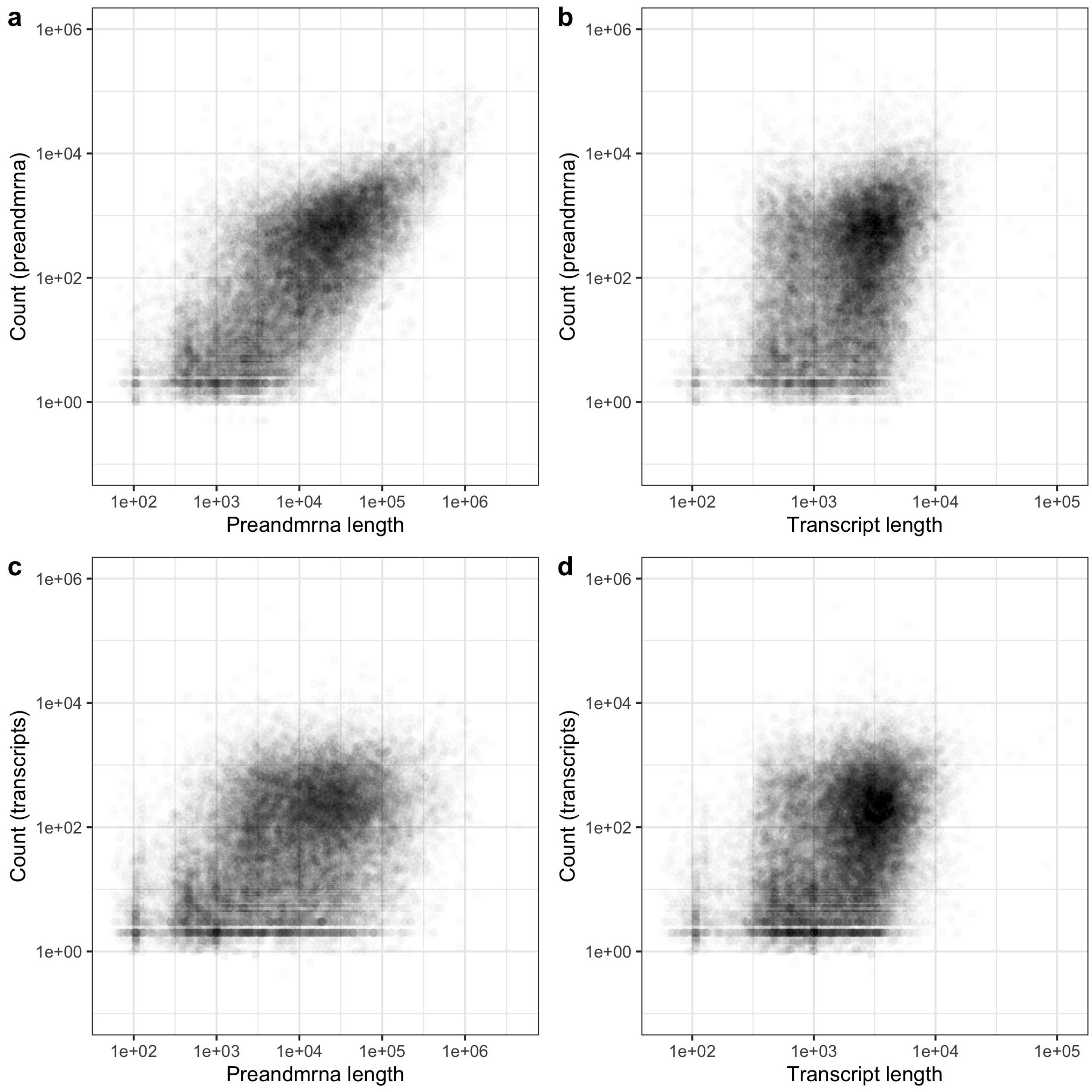

Supplementary Figure S12. Comparison of correlations between length and counts for the *preandmrna* and *transcripts* references. Each point is a different gene, with the length (base-10 log scale) on the x-axis and the counts (base-10 log scale) on the y-axis. The length is defined as either the full gene length with both exons and introns ('preandmrna' length) or the length with only exons ('transcripts' length). The counts are defined as the sum of reads across all nuclei under a given reference transcriptome (*preandmrna* or *transcripts*). Pearson's correlation coefficient (r) is calculated for each scatter plot. **(a)** 'preandmrna' length and *preandmrna* reference ($r = 0.68$) **(b)** 'transcript' length ($r = 0.37$) and *preandmrna* reference **(c)** 'preandmrna' length and *transcripts* reference ($r = 0.39$) **(d)** 'transcript' length and *transcripts* reference ($r = 0.38$).

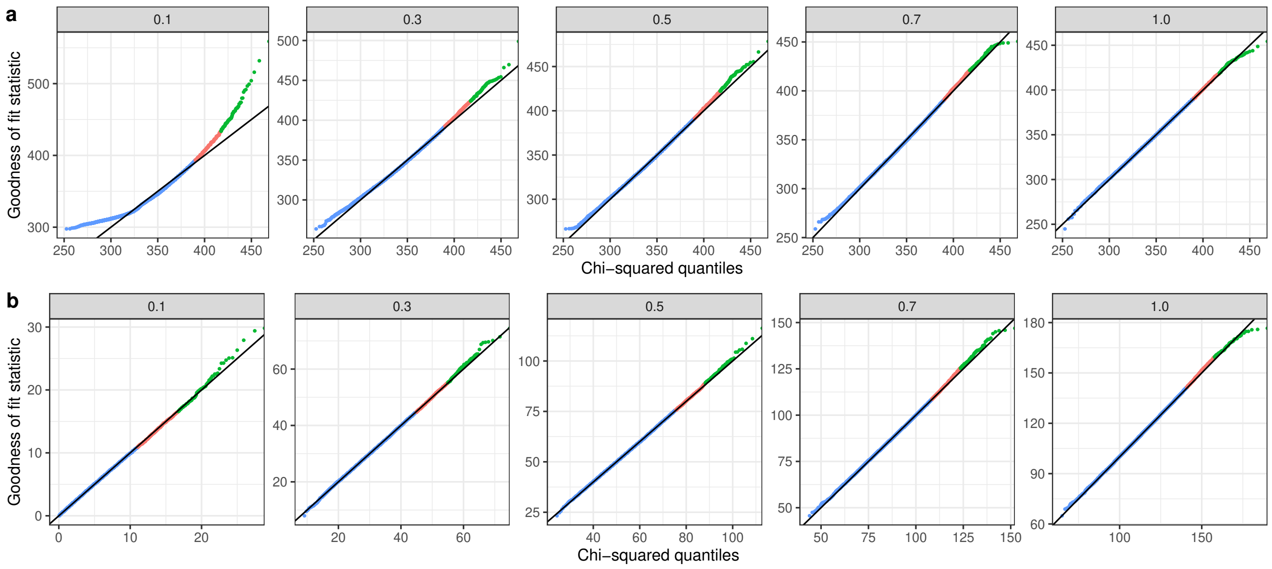

Supplementary Figure S13. Comparison of grouped versus ungrouped Poisson chi-squared tests. Using matrices of simulated Poisson counts with different μ parameters ($\mu = 0.1, 0.3, 0.5, 0.7, 1.0$ from left to right), the quantile-quantile plots from an **(a)** ungrouped Poisson chi-squared test and **(b)** grouped Poisson chi-squared test are compared.

Supplemental Tables

Reference transcriptome	Target sequences	Decoy sequence
transcripts	- spliced transcripts (mRNA): exonic regions of transcripts	genome
preandmrna	- spliced transcripts (mRNA): exonic regions of transcripts<br>- unspliced transcripts (pre-mRNA): full-length transcripts with both exonic and intronic regions	genome
introncollapse	- spliced transcripts (mRNA): exonic regions of transcripts<br>- introns "collapse": intronic regions extracted after collapsing all transcripts of a gene	genome
intronseparate	- spliced transcripts (mRNA): exonic regions of transcripts<br>- introns "separate": intronic regions extracted separately from each transcript isoform	genome

Supplementary Table S1. Summary of the reference transcriptome indices used in the quantification mapping tool. Reads are mapped to the target sequences in the reference transcriptome index. The reference transcriptome is augmented with decoy sequences, which mitigates the spurious mappings of reads that map better to the decoy sequences than the target sequences. In all reference transcriptomes, we provide the complete genome sequence as a decoy sequence to exclude reads coming from unannotated intergenic regions.

Reference transcriptome	Cortex	Cell type	Binomial BIC	Poisson BIC	NB BIC	G-S NB BIC
introncollapse	cortex_1	Astrocyte	993419	994418	991867	1193864
introncollapse	cortex_1	Endothelial	234996	235300	234517	330478
introncollapse	cortex_1	Excitatory neuron	6474847	6482392	6455055	6803449
introncollapse	cortex_1	Inhibitory neuron	3581353	3584667	3555615	3848241
introncollapse	cortex_1	Microglia	256956	257307	257095	358419
introncollapse	cortex_1	Oligodendrocyte	535866	536436	533698	683139
introncollapse	cortex_1	OPC	461936	462359	461431	607060
introncollapse	cortex_2	Astrocyte	2666923	2670728	2662550	2927818
introncollapse	cortex_2	Endothelial	542565	543105	539433	697640
introncollapse	cortex_2	Excitatory neuron	11711494	11728897	11683105	12043936
introncollapse	cortex_2	Inhibitory neuron	4539321	4546039	4521629	4829075
introncollapse	cortex_2	Microglia	188159	188338	187988	272574
introncollapse	cortex_2	Oligodendrocyte	1708922	1711333	1703927	1933407
introncollapse	cortex_2	OPC	1018338	1019629	1015129	1221247
intronseparate	cortex_1	Astrocyte	986476	987473	985101	1191448
intronseparate	cortex_1	Endothelial	235401	235705	235055	332604
intronseparate	cortex_1	Excitatory neuron	6389834	6397368	6370397	6719898
intronseparate	cortex_1	Inhibitory neuron	3547860	3551170	3522505	3819202
intronseparate	cortex_1	Microglia	255988	256339	256139	357593
intronseparate	cortex_1	Oligodendrocyte	526794	527363	524856	674236
intronseparate	cortex_1	OPC	459010	459434	458400	604232
intronseparate	cortex_2	Astrocyte	2681434	2685245	2677201	2949769
intronseparate	cortex_2	Endothelial	537555	538094	534376	693649
intronseparate	cortex_2	Excitatory neuron	11849547	11866993	11820394	12189465
intronseparate	cortex_2	Inhibitory neuron	4433085	4439780	4416641	4728909
intronseparate	cortex_2	Microglia	185954	186133	185790	269697
intronseparate	cortex_2	Oligodendrocyte	1712508	1714920	1707753	1946260
intronseparate	cortex_2	OPC	1014401	1015691	1011366	1221197
preandmrna	cortex_1	Astrocyte	970362	971355	969064	1179284
preandmrna	cortex_1	Endothelial	232215	232517	231903	328997
preandmrna	cortex_1	Excitatory neuron	6254964	6262467	6237851	6594304
preandmrna	cortex_1	Inhibitory neuron	3494344	3497646	3470333	3772494
preandmrna	cortex_1	Microglia	269287	269639	269516	377338
preandmrna	cortex_1	Oligodendrocyte	528615	529182	526990	679862
preandmrna	cortex_1	OPC	460119	460541	459689	609461
preandmrna	cortex_2	Astrocyte	2634890	2638678	2631783	2920277
preandmrna	cortex_2	Endothelial	545678	546217	542573	704570
preandmrna	cortex_2	Excitatory neuron	11726064	11743467	11699089	12081464
preandmrna	cortex_2	Inhibitory neuron	4422193	4428876	4406762	4728743
preandmrna	cortex_2	Microglia	182586	182763	182515	266519
preandmrna	cortex_2	Oligodendrocyte	1718763	1721169	1714387	1963111
preandmrna	cortex_2	OPC	1017102	1018389	1014503	1230944
transcripts	cortex_1	Astrocyte	526735	527646	526823	682108
transcripts	cortex_1	Endothelial	164601	164891	164089	236886
transcripts	cortex_1	Excitatory neuron	2730423	2737008	2730496	3032500
transcripts	cortex_1	Inhibitory neuron	1599691	1602611	1597088	1854117
transcripts	cortex_1	Microglia	134590	134902	134892	194633
transcripts	cortex_1	Oligodendrocyte	227464	227969	227209	313821
transcripts	cortex_1	OPC	220195	220570	220440	312221
transcripts	cortex_2	Astrocyte	1256567	1259985	1257531	1487992
transcripts	cortex_2	Endothelial	302298	302793	301089	407283
transcripts	cortex_2	Excitatory neuron	4782069	4797207	4786965	5126102
transcripts	cortex_2	Inhibitory neuron	1887368	1893215	1888198	2155326
transcripts	cortex_2	Microglia	87661	87822	87733	131771
transcripts	cortex_2	Oligodendrocyte	816042	818225	816071	1000431
transcripts	cortex_2	OPC	519215	520371	519276	675279

Supplementary Table S2. BIC log-likelihoods for each reference transcriptome, cortex, and cell type combination. The BIC log-likelihood are generally lowest for the negative binomial (NB) distribution or the binomial distribution, with similar BIC values for the Poisson distribution and higher values for G-S negative binomial, primarily due to the BIC penalty on the number of parameters. Negative binomial refers to a negative binomial distribution with one overdispersion parameter for all genes. G-S negative binomial refers to a negative binomial distribution with gene-specific overdispersion parameters.

References

[1] 10x Genomics. 5k Adult Mouse Brain Nuclei Isolated with Chromium Nuclei Isolation Kit, 7.0.0, Single Cell Gene Expression Dataset by Cell Ranger, 2022.

[2] J. Ding, X. Adiconis, S. K. Simmons, M. S. Kowalczyk, C. C. Hession, N. D. Marjanovic, T. K. Hughes, M. H. Wadsworth, T. Burks, L. T. Nguyen, J. Y. H. Kwon, B. Barak, W. Ge, A. J. Kedaigle, S. Carroll, S. Li, N. Hacohen, O. Rozenblatt-Rosen, A. K. Shalek, A.-C. Villani, A. Regev, and J. Z. Levin. Systematic comparison of single-cell and single-nucleus RNA-sequencing methods. *Nature Biotechnology*, 38(6):737–746, June 2020. ISSN 1546-1696. doi: 10.1038/s41587-020-0465-8.

[3] A. T. L. Lun, D. J. McCarthy, and J. C. Marioni. A step-by-step workflow for low-level analysis of single-cell RNA-seq data with Bioconductor. *F1000Res.*, 5:2122, 2016.

[4] J. C. Marioni, C. E. Mason, S. M. Mane, M. Stephens, and Y. Gilad. RNA-seq: an assessment of technical reproducibility and comparison with gene expression arrays. *Genome research*, 18(9):1509–1517, 2008.

[5] Z. Miao, M. S. Balzer, Z. Ma, H. Liu, J. Wu, R. Shrestha, T. Aranyi, A. Kwan, A. Kondo, M. Pontoglio, et al. Single cell regulatory landscape of the mouse kidney highlights cellular differentiation programs and disease targets. *Nature communications*, 12(1):2277, 2021.

[6] G. Schwarz. Estimating the Dimension of a Model. *The Annals of Statistics*, 1978. ISSN 0090-5364.

[7] F. W. Townes, S. C. Hicks, M. J. Aryee, and R. A. Irizarry. Feature selection and dimension reduction for single-cell RNA-Seq based on a multinomial model. *Genome Biol*, 20(1):295, 12 2019. doi: 10.1186/s13059-019-1861-6.

[8] M. N. Tran, K. R. Maynard, A. Spangler, L. A. Huuki, K. D. Montgomery, V. Sadashivaiah, M. Tippani, B. K. Barry, D. B. Hancock, S. C. Hicks, et al. Single-nucleus transcriptome analysis reveals cell-type-specific molecular signatures across reward circuitry in the human brain. *Neuron*, 109(19):3088–3103, 2021.

[9] H. Wickham. *ggplot2: Elegant Graphics for Data Analysis*. Springer-Verlag New York, 2016. ISBN 978-3-319-24277-4. URL https://ggplot2.tidyverse.org.

[10] G. R. Wood. Assessing goodness of fit for poisson and negative binomial models with low mean. *Communications in Statistics - Theory and Methods*, 31(11):1977–2001, 2002. doi: 10.1081/STA-120015014. URL https://doi.org/10.1081/STA-120015014.

[11] S. N. Wood. *Generalized Additive Models: An Introduction with R*. Chapman and Hall/CRC, 2 edition, 2017.

[12] H. Wu, Y. Kirita, E. L. Donnelly, and B. D. Humphreys. Advantages of Single-Nucleus over Single-Cell RNA Sequencing of Adult Kidney: Rare Cell Types and Novel Cell States Revealed in Fibrosis. *Journal of the American Society of Nephrology*, 30(1):23 LP – 32, 01 2019. URL http://jasn.asnjournals.org/content/30/1/23.abstract.
